# A membrane‐bound [NiFe]‐hydrogenase large subunit precursor whose C‐terminal extension is not essential for cofactor incorporation but guarantees optimal maturation

**DOI:** 10.1002/mbo3.1029

**Published:** 2020-03-16

**Authors:** Sven Hartmann, Stefan Frielingsdorf, Giorgio Caserta, Oliver Lenz

**Affiliations:** ^1^ Institut für Chemie Physikalische Chemie Technische Universität Berlin Berlin Germany

**Keywords:** chemolithotrophy, cofactor assembly, hydrogen, metalloenzyme, nickel, Tat transport

## Abstract

[NiFe]‐hydrogenases catalyze the reversible conversion of molecular hydrogen into protons end electrons. This reaction takes place at a NiFe(CN)_2_(CO) cofactor located in the large subunit of the bipartite hydrogenase module. The corresponding apo‐protein carries usually a C‐terminal extension that is cleaved off by a specific endopeptidase as soon as the cofactor insertion has been accomplished by the maturation machinery. This process triggers complex formation with the small, electron‐transferring subunit of the hydrogenase module, revealing catalytically active enzyme. The role of the C‐terminal extension in cofactor insertion, however, remains elusive. We have addressed this problem by using genetic engineering to remove the entire C‐terminal extension from the apo‐form of the large subunit of the membrane‐bound [NiFe]‐hydrogenase (MBH) from *Ralstonia eutropha*. Unexpectedly, the MBH holoenzyme derived from this precleaved large subunit was targeted to the cytoplasmic membrane, conferred H_2_‐dependent growth of the host strain, and the purified protein showed exactly the same catalytic activity as native MBH. The only difference was a reduced hydrogenase content in the cytoplasmic membrane. These results suggest that in the case of the *R. eutropha* MBH, the C‐terminal extension is dispensable for cofactor insertion and seems to function only as a maturation facilitator.

## Graphical abstract

The apo‐forms of [NiFe]‐hydrogenase large subunits are usually synthesized with a C‐terminal peptide extension that is proteolytically cleaved off upon incorporation of the catalytic metal center. Although to a limited amount, an artificially precleaved large subunit, which was deleted for the C‐terminal extension by genetic engineering, still received the active site components delivered by the dedicated maturation machinery. This suggests that the C‐terminal extension optimizes metal center incorporation, but is not essential for the formation of catalytically active [NiFe]‐hydrogenase.

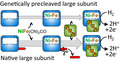

## 1 INTRODUCTION

The reversible conversion of molecular hydrogen (H_2_) into protons and electrons is catalyzed by the enzyme hydrogenase (Lubitz, Ogata, Rüdiger, & Reijerse, 2014). Hydrogenases are metalloenzymes involved in the energy metabolism of many microbial organisms (Vignais & Billoud, 2007), and their active sites occur in three different flavors, dependent on the metal composition of the active site. Accordingly, they are named [Fe]‐, [FeFe]‐, and [NiFe]‐hydrogenases (Greening et al., 2015; Lubitz et al., 2014; Schwartz, Fritsch, & Friedrich, 2013; Vignais & Billoud, 2007). [NiFe]‐hydrogenases represent probably the largest class, and their minimal H_2_‐converting module comprises a large subunit carrying the active site and a small, electron‐transferring subunit. The nickel and iron ions in the active site are coordinated to the protein backbone via four cysteine‐stemming thiolates. Two cysteines ligate both metals and therefore serve as bridging ligands. Furthermore, two cyanides (CN) and one carbon monoxide (CO) belong to the ligand sphere of the iron and keep the metal in a low‐spin Fe^2+^ state. Maturation and insertion of the NiFe(CN)_2_(CO) cofactor into the apo‐form of the large subunit require a sophisticated maturation machinery that consists of at least six auxiliary Hyp proteins (Böck, King, Blokesch, & Posewitz, 2006; Lacasse & Zamble, 2016). First, the Fe(CN)_2_(CO) moiety is assembled with the aid of the HypE and HypF proteins, which synthesize the cyanide ligands out of carbamoyl phosphate (Blokesch et al., 2004; Reissmann et al., 2003). The metabolic origin of CO under anaerobic conditions remains, however, unclear (Bürstel et al., 2011; Nutschan, Golbik, & Sawers, 2019), while under aerobic conditions, this diatomic ligand is derived from formyltetrahydrofolate (Bürstel et al., 2016; Schulz et al., 2020). Assembly takes place on a scaffold complex, consisting of the HypC and HypD proteins, from which the Fe(CN)_2_(CO) moiety is transferred to the apo‐large subunit (Bürstel et al., 2012; Stripp et al., 2013). Nickel is subsequently delivered to the active site with the help of the metallochaperones HypA and HypB. HypB was shown to feed HypA with nickel, which is compatible with a model in which HypA donates nickel to the large subunit (Lacasse, Summers, Khorasani‐Motlagh, George, & Zamble, 2019; Watanabe et al., 2015). The assumption that HypA delivers the active site nickel was recently supported by observations made for the apo‐large subunit of the cytoplasmic [NiFe]‐hydrogenase from *Thermococcus kodakarensis* that has been crystallized in a complex with the HypA protein (Kwon et al., 2018). Interestingly, the interaction of HypA with the flexible N‐terminus of the large subunit brought the chaperone in close vicinity of the still vacant active site. This was in fact a surprising result, because so far only the C‐terminal extension of the large subunit stood in the focus of [NiFe]‐hydrogenase maturation. The apo‐large subunit is usually synthesized with a C‐terminal extension comprising 3–68 amino acids (Greening et al., 2015), which is cleaved off by a specific endopeptidase once the complete NiFe site has been incorporated (Böck et al., 2006; Fritsch, Lenz, & Friedrich, 2013; Theodoratou, Huber, & Böck, 2005). Modifications of this C‐terminal extension, including amino acid exchanges, truncation (Theodoratou, Paschos, Mintz‐Weber, & Böck, 2000), or even complete removal (Massanz, Fernandez, & Friedrich, 1997; Senger, Stripp, & Soboh, 2017; Thomas, Muhr, & Sawers, 2015), by genetic engineering generally lead to the formation of inactive hydrogenase. Interestingly, while exchanges and truncations revealed a premature large subunit that was unable to form a complex with the small subunit, genetic removal of the entire extension allowed the formation of hydrogenase with canonical subunit composition. This phenomenon has been observed for the soluble, NAD^+^‐reducing [NiFe]‐hydrogenase of *R. eutropha* (Massanz et al., 1997) and, more recently, for membrane‐bound [NiFe]‐hydrogenase 2 (Hyd‐2) from *Escherichia coli* (Thomas et al., 2015). In both cases, however, the large subunit was at least devoid of nickel (Massanz et al., 1997). Nickel‐free Hyd‐2 was also shown to lack the CN and CO ligands of the Fe(CN)_2_(CO) moiety, which are easily detectable by infrared spectroscopy (Senger et al., 2017). These observations suggest an essential role of the C‐terminus in active site maturation.

However, it has to be stressed at this point that numerous [NiFe]‐hydrogenases are naturally devoid of a C‐terminal extension, yet they seem to employ the canonical maturation machinery to acquire a NiFe active site. Prominent members are the H_2_‐sensing hydrogenases (belonging to groups 2b and 2d according to (Greening et al., 2015)), CO dehydrogenase‐associated hydrogenases (group 4c), Ech‐type hydrogenases (group 4e), and certain so far uncharacterized hydrogenases (group 4g) (Greening et al., 2015). The parallel occurrence of [NiFe]‐hydrogenase large subunits with and without C‐terminal extension leaves the importance of the C‐terminal extension in the maturation of [NiFe]‐hydrogenases ambiguous.

To obtain further information, we investigated the role in maturation of the C‐terminal extension of the large subunit of the membrane‐bound [NiFe]‐hydrogenase from the model H_2_ oxidizer *R. eutropha* H16. This *β*‐proteobacterium harbors four different O_2_‐tolerant [NiFe]‐hydrogenases that are either directly or indirectly involved in energy generation from the controlled combustion of H_2_ with O_2_ (Lenz, Lauterbach, Frielingsdorf, & Friedrich, 2015). Three of them, the soluble cytoplasmic NAD^+^‐reducing [NiFe]‐hydrogenase, the membrane‐bound [NiFe]‐hydrogenase (MBH), and the actinobacterial‐like [NiFe]‐hydrogenase, harbor large subunits whose apo‐forms carry C‐terminal extensions. The large subunit of the H_2_‐sensing regulatory hydrogenase (RH), by contrast, is devoid of a C‐terminal extension (Kleihues, Lenz, Bernhard, Buhrke, & Friedrich, 2000), although being equipped with a canonical NiFe(CN)_2_(CO) center (Bernhard et al., 2001), which is incorporated by the Hyp machinery of *R. eutropha* (Buhrke, Bleijlevens, Albracht, & Friedrich, 2001).

The basic hydrogenase module of the MBH of *R. eutropha* consists of the large subunit HoxG carrying the NiFe(CN)_2_(CO) cofactor and the small subunit HoxK comprising three iron–sulfur (Fe‐S) clusters (Fritsch et al., 2011). Upon incorporation of the NiFe(CN)_2_(CO) cofactor into HoxG by the Hyp machinery, the specific endopeptidase HoxM cleaves off the C‐terminal extension. Subsequently, the mature subunits form the HoxGK heterodimer, which is then translocated via the Tat pathway through the cytoplasmic membrane and attached to a membrane‐integral cytochrome *b* (Frielingsdorf, Schubert, Pohlmann, Lenz, & Friedrich, 2011; Schubert, Lenz, Krause, Volkmer, & Friedrich, 2007). Deletion of the gene encoding the MBH‐specific endopeptidase HoxM results in the accumulation of a HoxG preform still carrying the C‐terminal extension (Bernhard, Schwartz, Rietdorf, & Friedrich, 1996; Hartmann et al., 2018).

In this study, we deleted the C‐terminal extension of HoxG by genetic engineering and show that the resulting truncated version of HoxG still receives a NiFe(CN)_2_(CO) cofactor and forms, together with the corresponding HoxK subunit, catalytically active MBH.

## 2 MATERIALS AND METHODS

### 2.1 Genetic constructions

All bacterial strains and plasmids used in theis study are listed in Table 1. The sequence encoding the C‐terminal extension of *hoxG* (amino acids 604–618 of HoxG, (Fritsch et al., 2011)) was eliminated by PCR amplification using the primers SFP43 5′‐AAGAATGTATACGTGCCAGACGTG‐3′ and SFP44 5′‐ACTAAGCTTTTAGTGAGTCGAACACGCCAGAC‐3′ using pJH5415 as template. The PCR product was digested with AccI/HindIII and ligated into AccI/HindIII‐cut pJH5415 yielding pSF8.14. pSF8.14 was digested with SpeI/XbaI, and the resulting 3595‐bp fragment was ligated into XbaI‐cut pEDY309 yielding pSF10.8. This plasmid was transformed into *E. coli* S17–1 (AK2429) for subsequent conjugative transfer to *R. eutropha* strain HF1063 yielding strain HP9. The wild‐type control strain was generated by digesting pJH5415 with SpeI/XbaI and transfer of the resulting fragment into pEDY309, yielding pJH5437. Plasmid pJH5437 was transferred by conjugation into strain HF1063, yielding strain HP3.

**Table 1:** Strains and plasmids used in this study

Strain/plasmid	Relevant characteristics	Comments/reference
*Ralstonia eutropha*		
HF388	Δ*hoxH*	Bernhard et al., (1996)
HF649	pGE636 in HF631, *hoxK*‐Strep‐Tag II	Schubert et al., (2007)
HF1063	Derivative of HF388 carrying the in‐frame deletions Δ*hoxG*, Δ*hoxK*, Δ*hoxH*, and Δ*hoxC*	This study
HP3	Strain HF1063 carrying pJH5437; P_MBH_‐*hoxK*_Strep_‐*hoxG;* MBH^+^,SH^–^, RH^–^	This study
HP9	Strain HF1063 carrying pSF10.8; P_MBH_‐*hoxK*_Strep_‐*hoxG* ^ΔAA604−618^; MBH^+^,SH^–^, RH^–^	This study
*Escherichia coli*		
S17–1	Tra^+^ *recA*, *pro thi*, *hsdR chr*:RP4–2	Simon, Priefer, & Pühler, (1983)
JM109	F´ *traD36 lacI^q^*, Δ(*lacZ*) *M15 proA^+^B^+^*/*e14* ^–^ (*McrA^–^*) Δ(*lac‐proAB*) *thi gyrA96* (Nal*^r^*) *endA1 hsdR17* (rk^–^ mk^+^) *relA1 supE44 recA1*	Yanisch‐Perron, Vieira, & Messing, (1985)
Plasmids		
LITMUS 28	Ap^r^, *lacZ′*, ColE1 *ori*	New England Biolabs
pEDY309	Tc^r^, RK2 *ori*, Mob^+^	Kleihues et al., (2000)
pJH5415	P_MBH_‐*hoxK_Strep_‐hoxG* in LITMUS 28	(formerly pCH#5415) Frielingsdorf et al., (2014)
pJH5437	P_MBH_‐*hoxK_Strep_‐hoxG* in pEDY309	This study
pSF8.14	P_MBH_‐*hoxK_Strep_‐hoxG* ^ΔAA604−618^ in LITMUS 28	This study
pSF10.8	P_MBH_‐*hoxK_StrepTagII_‐hoxG* ^ΔAA604−618^ in pEDY309	This study

Strain HF1063 was generated from HF388 by the introduction of isogenic deletions of *hoxC* and *hoxK‐hoxG* following published procedures (Lenz, Lauterbach, & Frielingsdorf, 2018).

### 2.2 Media composition and cell cultivation

*Ralstonia eutropha* strains HF649, HF1063, HP3, and HP9 were cultivated in FGN_mod_ medium as described elsewhere (Hartmann et al., 2018; Lenz et al., 2018). Cells were harvested by centrifugation (11,500 *g*, 4°C, 12 min), and the resulting cell pellet was frozen in liquid nitrogen and stored at − 80°C. Lithoautotrophic cultivation in liquid and on agar‐solidified minimal medium devoid of organic carbon sources was carried out as described previously (Lenz et al., 2018) with the exception that a gas atmosphere of 10% (v/v) CO_2_, 4% (v/v) H_2_, 10% (v/v) O_2,_ and 76% (v/v) N_2_ was used. Main cultures were inoculated with a preculture to an initial OD_436 nm_ of 0.1 and shaken at 120 rpm and 30°C for 16 days. Agar plates were incubated for 6 days at 30°C.

### 2.3 Protein extract preparation, polyacrylamide gel electrophoresis, and immunological analysis

To analyze the MBH subunit content in different cellular protein fractions, cell pellets were resuspended in 3 ml (per gram wet weight) of resuspension buffer (50 mM K_2_HPO_4_/KH_2_PO_4_, pH 7.3, 150 mM NaCl) containing cOmplete EDTA‐free protease inhibitor mixture (Roche Diagnostics) and DNase I (Roche Diagnostics). The resuspended cells were disrupted in a chilled French pressure cell at 124.11 MPa. This procedure resulted in whole‐cell lysate (“lysate” sample). Unbroken cells and cell debris were subsequently sedimented by centrifugation (4,000 *g*, 4°C, 20 min), yielding an emulsion composed of membranes and soluble proteins. The emulsion was ultracentrifuged (100,000 *g*, 4°C, 1 hr), yielding a dark‐brown membrane pellet and a brownish liquid supernatant (“soluble extract” sample). The membrane pellet was washed by homogenization with a Potter‐Elvehjem homogenizer in 10 ml of resuspension buffer (per gram, wet weight, of the membrane) containing protease inhibitor cocktail. The suspension was then ultracentrifuged (100,000 *g*, 4°C, 35 min), yielding clean membranes as a pellet. Its resuspension in resuspension buffer yielded the sample “membrane”. Sodium dodecyl sulfate–polyacrylamide gel electrophoresis (SDS‐PAGE) (Laemmli, 1970) was used for protein separation, which was followed by Western blot analysis (Towbin, Staehelin, & Gordon, 1979). Proteins in gels were either stained with Coomassie brilliant blue G‐250 (Diezel, Kopperschläger, & Hofmann, 1972) or transferred to a nitrocellulose membrane (BioTrace, Pall Corp.) using a fast semidry transfer buffer.(Garić et al., 2013) Polyclonal antibodies raised against the MBH large subunit (anti‐HoxG, (Bernhard et al., 1996)) in combination with a goat anti‐rabbit secondary antibody (coupled with alkaline phosphatase, Dianova) were used for detection of HoxG. Protein concentrations were determined with the Pierce BCA Protein Assay Kit (Thermo Scientific), using bovine serum albumin as standard.

### 2.4 MBH purification

Purification of the MBH*_Strep_* variants derived from *R. eutropha* HP3, HP9, and HF649 was carried out as described previously (Goris et al., 2011; Lenz et al., 2018). The protein samples were frozen in liquid nitrogen and stored at − 80°C.

### 2.5 H_2_ oxidation activity assay

H_2_‐mediated reduction of methylene blue was determined spectrophotometrically as previously described (Lenz et al., 2018) using a Cary50 UV–Vis spectrophotometer (Varian, Agilent). The specific activity was given in Units (U) per mg of protein, where 1 U corresponds to the turnover of 1 µmol H_2_ per minute.

### 2.6 Fourier‐transform infrared (FTIR) spectroscopy

A volume of 10 µl with MBH protein concentration of 0.3 mM was transferred into a gas‐tight, homemade, temperature‐controlled (10°C), transmission cell equipped with sandwiched CaF_2_ windows and a Teflon spacer (optical path length of 50 µm). Spectra with 2 cm^‐1^ resolution were recorded on a Tensor 27 Fourier‐Transform spectrometer (Bruker) equipped with a liquid N_2_‐cooled mercury cadmium telluride (MCT) detector. For a single spectrum, 200 scans were averaged. The sample compartment was purged with dried air. A buffer spectrum was taken to calculate the corresponding absorbance spectra. Bruker OPUS software 7.5 was used for data analysis.

## 3 RESULTS

Previous studies revealed that the genetic removal of the C‐terminal extension of the large subunit results in hydrogenase devoid of a functional NiFe active site (Massanz et al., 1997; Thomas et al., 2015). To test whether the absence of the C‐terminal extension of the membrane‐bound hydrogenase (MBH) of *R. eutropha* also leads to inactive protein, we deleted the sequence encoding the amino acid residues Val604‐Arg618 of the HoxG subunit. The deletion resulted in a preprocessed HoxG subunit, henceforth designated as HoxG^proc^, terminating with residue His603 that also represents the very last residue upon cleavage of the native subunit with the protease HoxM (Fritsch et al., 2011). The corresponding *hoxG* ^proc^ gene was cloned together with *hoxK*_Strep_, which encodes the C‐terminally Strep‐tag II‐tagged HoxK subunit of the MBH, onto the broad‐host‐range vector pEDY309. The native MBH promoter controlled the expression of both genes on the resulting plasmid pSF10.8.

**FIGURE 1:**
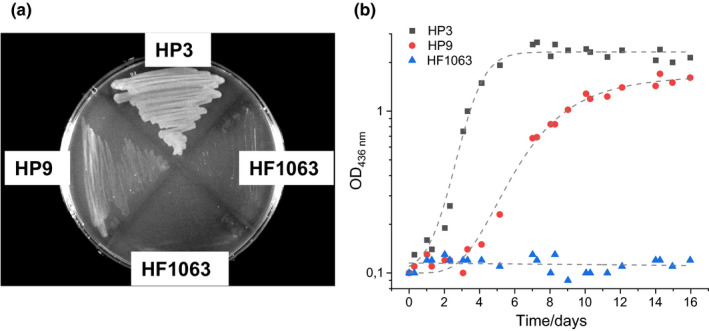
The C‐terminal extension of HoxG is dispensable for MBH‐driven chemolithoautotrophic growth. *Ralstonia eutropha* strains HF1063, HP3, and HP9 were cultivated in minimal medium under a gas atmosphere of 10% (v/v) CO_2_, 4% (v/v) H_2_, 10% (v/v) O_2,_ and 76% (v/v) N_2_. (a) Growth after 6 days at 30°C on Bacto agar‐solidified medium. (b) Growth of *R. eutropha* HF1063 (blue triangles), HP3 (black squares), and HP9 (red circles) in liquid cultures. Optical densities were measured at 436 nm (OD_436 nm_)

First, we tested the impact of the absence of the C‐terminal extension on the capability of recombinant *R. eutropha* cells to grow chemolithoautotrophically in a minimal medium under a gas mixture of H_2_, CO_2_ and O_2_. We transferred plasmids pSF10.8 and pJH5437, the latter encodes native MBH, to *R. eutropha* HF1063, which—owing to in‐frame deletions within the genes *hoxG* and *hoxH*—is incapable in chemolithoautotrophic growth. The *hoxH* gene encodes the large subunit of the soluble, NAD^+^‐reducing hydrogenase (SH) of *R. eutropha*. Both the MBH and SH can mediate independently chemolithoautotrophic growth of *R. eutropha* (Hogrefe, Römermann, & Friedrich, 1984). Thus, bare *R. eutropha* HF1063 served as negative control, and *R. eutropha* HF1063(pJH5437), designated as HP3, as the corresponding positive control. *Ralstonia eutropha* HF1063(pSF10.8) was named HP9*.* The three *R. eutropha* derivatives were grown on minimal medium devoid of organic carbon sources under a gas atmosphere of 10% (v/v) CO_2_, 4% (v/v) H_2_, 10% (v/v) O_2,_ and 76% (v/v) N_2_. The observed growth patterns on solid medium (Figure 1a) and in liquid culture (Figure 1b) were coherent. *Ralstonia eutropha* HP3 grew well, while HF1063 did not grow at all. Although less well than the positive control, also *R. eutropha* HP9 showed H_2_‐driven chemolithotrophic growth. These results clearly demonstrate that the C‐terminal peptide extension of HoxG is dispensable for the formation of catalytically active MBH.

**FIGURE 2:**
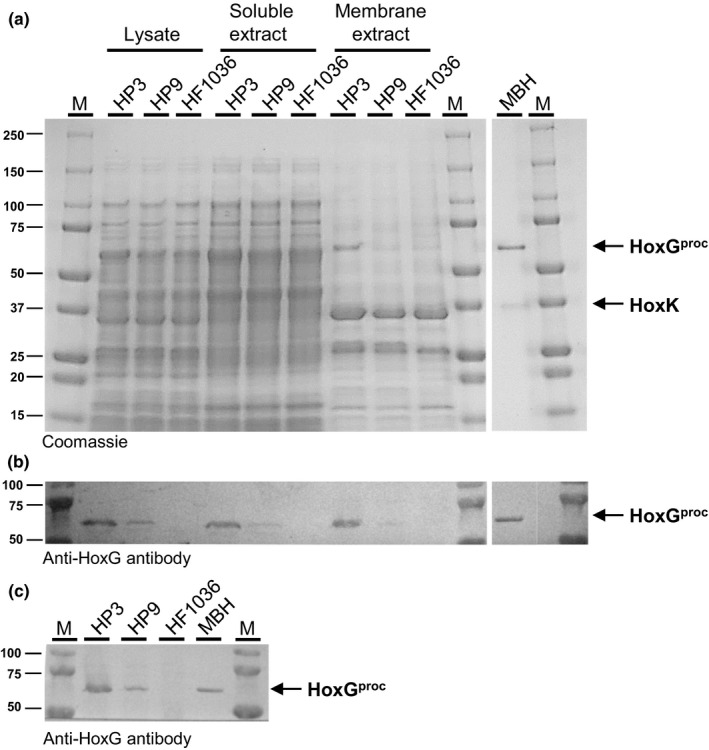
Genetic removal of the C‐terminal extension of HoxG leads to a decreased MBH content in the cytoplasmic membrane. Heterotrophically grown cells of *R. eutropha* HP3, HP9, and HF1063 were lysed (lysate) and subsequently fractionated into soluble extract as well as membrane extract (membrane). Native MBH purified from strain *R. eutropha* HF649 served as positive control. Samples were analyzed by SDS‐PAGE and immunological detection. (a) Coomassie‐stained SDS‐PAGE gel; (b) and (c) Western blots developed using anti‐HoxG antibodies. Protein amounts in (a) and (b): lysate—20 µg, soluble extract—20 µg, membrane—5 µg, and MBH—0.5 µg. The labels of the lanes in (b) are the same as in (a). In (c), 20 µg of membrane proteins and 0.3 µg purified MBH were loaded. M: Marker (Precision Plus Protein™ Dual Color Standard 10–250 kDa from Bio‐Rad)

To examine whether the missing C‐terminus of HoxG affects the amount of MBH molecules in the membrane, we performed Western immunoblot analyses. Cells of *R. eutropha* HF1063, HP3, and HP9 were grown in hydrogenase‐derepressing minimal medium with fructose and glycerol as the carbon and energy sources. At an OD_436 nm_ of approximately 12, the cells were collected by centrifugation, and whole‐cell lysates, soluble extracts, and membrane fractions were prepared (Figure 2). Proteins were separated on an SDS‐PAGE gel, and the HoxG protein was detected with a polyclonal HoxG‐specific antibody. The immunoblots shown in Figure 2b,c clearly revealed that HoxG^proc^ is significantly less abundant than native HoxG. We observed similar HoxG^proc^/HoxG ratios in all three cell fractions, indicating that the low HoxG^proc^ content in the membrane was not caused by impaired Tat transport. As expected, no HoxG signal was detected in cell fractions of HF1063. We conclude that the amount of MBH inserted into the membrane is significantly lower in the membrane fraction of HP9 when compared to that of HP3. Thus, the slow chemolithoautotrophic growth of *R. eutropha* HP9 (Figure 1) is presumably caused by the low MBH content in the cytoplasmic membrane.

According to the current model, the C‐terminus of the large subunit is cleaved off only if nickel had been inserted properly into the active site (Böck et al., 2006). In 2015, Sawers and coworkers have challenged this model. They observed that the genetically processed large subunit of *E. coli* Hyd2 lacks the native NiFe cofactor but forms a complex with the small subunit. The resulting inactive Hyd2 was even accepted by the Tat translocation apparatus and appropriately inserted into the cytoplasmic membrane (Thomas et al., 2015). Therefore, we analyzed the catalytic activity and the cofactor content of the MBH^proc^ purified from the membrane fraction of *R. eutropha* HP9 and compared the results with those of *R. eutropha* HP3, synthesizing native MBH. The MBH yield from *R. eutropha* HP9 and HP3 was (115 ± 28) µg and (208 ± 11) µg, respectively, of protein per gram of cells (wet weight). Thus, membranes of strain HP9 had a ~45% lower MBH content than strain HP3, which is in line with the Western blot results (Figure 2b and c). Both MBH versions showed, however, identical specific activities for H_2_‐mediated methylene blue reduction, with (87.8 ± 3.4) U/mg for native MBH and (88.5 ± 4.7) U/mg for MBH^proc^. Thus, despite the genetic removal of the C‐terminal extension, the NiFe active site of MBH^proc^ seemed to be correctly assembled.

**FIGURE 3:**
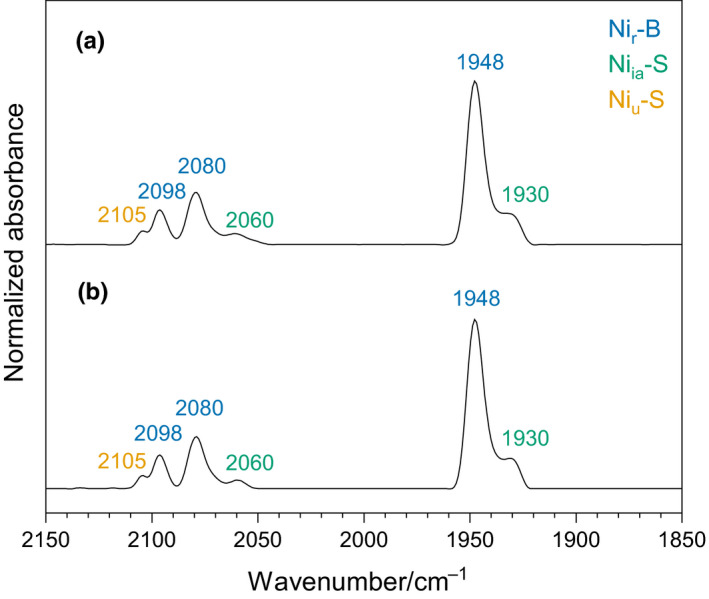
Native MBH and MBH^proc^ are populated to the same extent with a canonical NiFe active site. Infrared absorbance spectra of aerobically purified native MBH from *R. eutropha* HP3 (a) and MBH^proc^ from *R. eutropha* HP9 (b). Both as‐isolated proteins (pH 7.1) resided mainly in the Ni_r_‐B state (blue) with minor contributions of the Ni_ia_‐S (green) and the Ni_u_‐S (orange) states. The dominant Ni_r_‐B peaks superpose unassigned contributions of Ni_ia_‐S and Ni_u_‐S. The spectra were normalized to the protein concentration

To investigate the integrity of the active site further, we performed infrared (IR) spectroscopy, which probes the C≡O and C≡N stretching vibrations associated with the CO and CN^–^ ligands of the NiFe site. These vibrations are very sensitive to structural and redox modifications of the active site (Bagley, Duin, Roseboom, Albracht, & Woodruff, 1995). The resulting IR spectra of as‐isolated, oxidized native MBH and MBH^proc^ are shown in Figure 3. To obtain quantitative information on the loading of the proteins with the NiFe cofactor, the spectra were normalized based on the intensity of the amide II band, which is proportional to the protein concentration. Both spectra were almost identical to that of aerobically purified MBH (Goris et al., 2011) and dominated by absorption bands assigned to the Ni_r_‐B state of the active site (Figure 3, blue labels), characterized by a hydroxo ligand bridging the Ni and Fe ions. Furthermore, minor contributions of the inactive Ni_ia_‐S (Figure 3, green labels) and the unready Ni_u_‐S state (Figure 3, yellow labels) were observed (Saggu et al., 2009). Both the positions and the intensities of the IR bands were nearly identical for native MBH and MBH^proc^. Thus, both MBH versions were identically populated with a canonical NiFe active site.

## 4 DISCUSSION

The MBH^proc^ of *R. eutropha* is the first example of a [NiFe]‐hydrogenase that is equipped with a NiFe catalytic center, although the C‐terminal extension of the large subunit was genetically removed. In fact, the purified MBH^proc^ protein was indistinguishable from native MBH with respect to the active site architecture and catalytic activity. Thus, the C‐terminal extension of the large subunit is not essential for Hyp protein‐mediated insertion of the NiFe cofactor. Nevertheless, removal of the C‐terminal extension led to significantly lowered MBH levels in the membrane. Thus, our results indicate that the C‐terminal extension optimizes maturation efficiency. Notably, there was no indication for apo‐MBH, that is, MBH without NiFe cofactor, in our membrane‐derived protein preparation. This is in clear contrast to previous reports for *E. coli* Hyd‐2, where catalytically inactive hydrogenase complexes were identified that contained genetically processed, but NiFe cofactor‐free large subunits (Massanz et al., 1997; Senger et al., 2017; Thomas et al., 2015). It has been convincingly shown that the Fe‐S cluster‐containing hydrogenase small subunit, which is equipped with the Tat leader peptide, becomes transported through the cytoplasmic membrane only in complex with the large subunit (Rodrigue, Chanal, Beck, Müller, & Wu, 1999; Schubert et al., 2007). The results by Thomas et al. suggest that the mere attachment of the large subunit to the small subunit elicits the signal to initialize the Tat‐dependent membrane transport of *E. coli* Hyd‐2, irrespective of the presence of the NiFe cofactor (Thomas et al., 2015). Assuming invariant Tat mechanisms in *E. coli* and *R. eutropha*, the absence of immature MBH in *R. eutropha* membranes signifies an upstream control step that prevents the complex formation of mature small subunits with immature but processed large subunits. Alternatively, NiFe cofactor‐free hydrogenase complexes may be subjected to proteolysis. The latter mechanism is rather unlikely, because the genetically processed large subunit of the soluble, NAD^+^‐reducing [NiFe]‐hydrogenase, SH, of *R. eutropha* forms a complex with the remaining hydrogenase subunit, although no nickel had been inserted into the active site (Massanz et al., 1997). Thus, the small subunits themselves might reject processed premature large subunits lacking a complete NiFe cofactor, and this capability seems not to be uniformly distributed among all hydrogenases. While the SH small subunit obviously cannot distinguish between processed mature and immature large subunits (Massanz et al., 1997), those of the MBH and the regulatory [NiFe]‐hydrogenase (RH) obviously can. In fact, the RH belongs to the subclass of hydrogenases whose apo‐large subunits natively lack the C‐terminal extension, but are recognized by the canonical Hyp machinery that inserts the canonical NiFe cofactor (Buhrke et al., 2001). Purified RH protein consisted of the iron–sulfur cluster‐containing small subunit and large subunit that was stoichiometrically loaded with nickel (Buhrke et al., 2005), indicating an intrinsic proofreading mechanism that prevents the complex formation of premature subunits. The same seems to be true for MBH^proc^.

**FIGURE 4:**
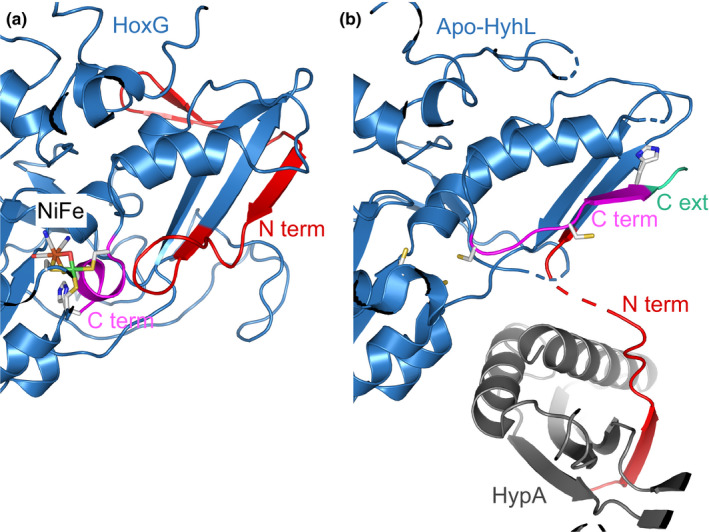
Structural comparison of immature *T. kodakarensis* HyhL in complex with the maturase HypA and the mature HoxG subunit of the MBH from *R. eutropha*. The protein backbones of the large subunits are depicted in blue with red N‐termini and magenta C‐termini (cartoon representation). The structural models with the PDB codes 4IUC (mature HoxG) (Frielingsdorf et al., 2014) and 5YXY (preform of apo‐HyhL (Kwon et al., 2018)) were used. (a) The active site, including the coordinating cysteines and the C‐terminal histidine of mature HoxG, are shown as stick models. The C‐terminal extension is not visible, as it has been cleaved off. Note that the N‐terminus (red) adapts to a β‐sheet domain of the main protein, and one of the N‐terminal β‐strands is located at the position, which is occupied by a β‐strand structure of the C‐terminal domain in apo‐HyhL (b). (b) The C‐terminal extension (C ext) of apo‐HyhL, which is cleaved off upon NiFe cofactor insertion, is shown in mint. The red N‐terminus (the region between the two ends of the red line is structurally unresolved and depicted as a broken line) protrudes from the globular protein and forms a complex with HypA (gray). The four conserved cysteines that coordinate the NiFe(CN)_2_(CO) cofactor are represented as sticks. The terminal histidine residue (shown as sticks), which lies directly in front of the cleavage site of the C‐terminal extension, was not resolved and therefore was modelled computationally into the structure using PyMol (The PyMOL Molecular Graphics System, Version 2.2.0 Schrödinger, LLC)

The fact that both HoxG^proc^ and the RH large subunit properly receive the NiFe(CN)_2_(CO) cofactor implies that the C‐terminal extension is not required for interaction of the Hyp apparatus with the large subunit. This conclusion is in contrast to in vitro experiments showing the interaction of the apo‐form of the large subunit of *E. coli* Hyd‐2 with the Hyp protein delivering the Fe(CN)_2_(CO) unit of the active site was abolished in the absence of the C‐terminal extension (Senger et al., 2017). However, there are strong indications that rather the N‐terminus of the large subunit is involved in the interaction with the Hyp machinery (Albareda, Buchanan, & Sargent, 2017; Kwon et al., 2018; Pinske, Thomas, Nutschan, & Sawers, 2019; Thomas et al., 2015). Just recently, the crystal structure of apo‐HyhL, the large subunit of the [NiFe]‐hydrogenase from *T. kodakarensis*, in complex with the nickel‐inserting maturase HypA has been resolved (Kwon et al., 2018). Interestingly, the structure of the N‐terminus of HyhL adopted a different localization/conformation than the N‐termini of large subunit structures of mature [NiFe]‐hydrogenases, such as *R. eutropha* MBH (Figure 4). In fact, the C‐terminal extension of HyhL occupies parts of the position of the N‐terminus in the mature structure, forcing the N‐terminus in another direction (Kwon et al., 2018). As a consequence, the N‐terminus acts like a “crane arm,” which brings the HypA protein close to the active site cavity where it can deliver the nickel ion (Figure 4). Nickel incorporation presumably leads to a dramatic conformational change in the C‐terminal extension that upon cleavage moves close to the active site cavity, which unblocks the dedicated position of the N‐terminus of the mature protein (Kwon et al., 2018). Therefore, a major role of the C‐terminal extension might be providing indirectly the N‐terminus with sufficient flexibility to interact with the Hyp machinery. A role of the C‐terminal extension in enhancing structural flexibility to facilitate the interaction with Hyp proteins has also been proposed (Albareda, Pacios, & Palacios, 2019; Pinske et al., 2019). A function as a maturation facilitator would also explain why the removal of the C‐terminal extension does not necessarily lead to immature hydrogenase. In case of *R. eutropha* MBH^proc^, the assembly process seemed to be just less efficient, resulting in a reduced amount of fully active MBH in the cytoplasmic membrane. It should be mentioned, however, that the genetic removal of the extension might result in reduced stability of the large subunit before it becomes equipped with NiFe(CN)_2_(CO) cofactor and oligomerizes with the small subunit. To clarify the overall necessity of the C‐terminal extension, more large subunits need to be tested for their capacity to tolerate the absence of the C‐terminal extension in the course of NiFe cofactor insertion.

## CONFLICT OF INTERESTS

None declared.

## AUTHORS' CONTRIBUTION

Sven Hartmann: Conceptualization (equal); Investigation (lead); Writing‐original draft (lead). Stefan Frielingsdorf: Conceptualization (equal); Investigation (supporting); Project administration (equal); Writing‐review & editing (equal). Giorgio Caserta: Investigation (supporting); Writing‐original draft (supporting). Oliver Lenz: Conceptualization (supporting); Funding acquisition (lead); Project administration (equal); Writing‐review & editing (equal). 

## ETHICS STATEMENT

None required.

## Data Availability

All data are provided in full in the results section of this paper.
